# Verification of Normality in the Source Intensity Detection Rate for Batch Assay Using a Well-Type Ionization Chamber

**DOI:** 10.7759/cureus.96627

**Published:** 2025-11-11

**Authors:** Masato Takanashi, Isao Kuroda, Shinji Sugahara, Masumi Kawaguchi, Masataka Hoshina, Masaya Noguchi, Koichi Masuda

**Affiliations:** 1 Department of Radiology and Radiation Oncology, Tokyo Medical University Ibaraki Medical Center, Ibaraki, JPN; 2 Department of Urology, Tokyo Medical University Ibaraki Medical Center, Ibaraki, JPN

**Keywords:** 125i seed brachytherapy, batch assay, brachytherapy, iodine-125, low-dose-rate brachytherapy, prostate cancer, single-seed assay, well-type ionization chamber

## Abstract

The American Association of Physicists in Medicine (AAPM) advocates for the importance of source intensity verification at the facility of use. At our facility, we have continuously performed source intensity verification on all blister packs delivered since initiating 125I seed brachytherapy. The single-seed assay recommended by the AAPM is difficult to implement domestically. As an alternative method, batch assays using an ionization chamber or a well-type ionization chamber to measure multiple seeds simultaneously have been proposed. Our facility calculates the source strength detection rate (detection rate) from measurements using a well-type ionization chamber to verify the accuracy of source strength. Understanding the characteristics of these measurements is crucial for their proper handling. Previous studies have reported verifications for the model STM1251 (C.R. Bard, Murray Hill, NJ) and the OncoSeed model 6711 (General Electric Healthcare, Barrington, IL). However, to our knowledge, there are no reports verifying whether the detection rate for the TheraAgX100 (Theragenics Corporation, Buford, GA) used at our facility is normally distributed. The purpose of this study is to verify the normality of detection rates based on data accumulated to date. The measurement subjects were the five blister packs with the highest number of deliveries among the delivered TheraAgX100 blister packs, totaling 262 cases. For all of these, the detection rate was calculated from the measurements obtained using the CRC-15R well-type ionization chamber (Capintec Corp., Ramsey, NJ). Normality was verified using Statistical Product and Service Solutions (SPSS, version 29; IBM SPSS Statistics for Windows, Armonk, NY). The Shapiro-Wilk test was used for the test, with a significance level of 0.05. The results of the normality test showed a Shapiro-Wilk test significance probability of 0.623, confirming that the detection rate follows a normal distribution. For batch assays, the nominal value must be calculated from the accumulated measurements at the facility. In other words, without confirming the normality of the detection rate, it is unclear whether the mean or median should be adopted as the nominal value. This study demonstrated that, for the combination of radiation sources and dosimeters at this facility, the detection rate follows a normal distribution. Therefore, it is valid to use either the mean or the median of the detection rate as the nominal value. The guidelines describe the response when batch assay results deviate from the nominal value. Specific control limits, such as tolerance limits and intervention limits, are indicated. For batch assays, the nominal value must be calculated based on the cumulative detection rate at the facility. If the detection rate does not follow a normal distribution, using the mean or median from the batch assay as the nominal value is not appropriate. This study demonstrated the validity of using the mean or median of the cumulative detection rate as the nominal value. We plan to verify this approach for blister packs containing different numbers of units. Furthermore, since the shape of the radiation source varies by product, re-evaluation is necessary when new products are introduced. Understanding the characteristics of the acquired measurements and applying this knowledge to clinical practice is a critical responsibility of medical physicists in charge of quality assurance.

## Introduction

Prostate cancer is one of the most common malignant tumors in men, and its incidence is increasing worldwide [1,2]. In Japan, 125I seed brachytherapy was introduced in 2003 and has been used to treat many patients [3]. One advantage of 125I seed brachytherapy is the low incidence of reported adverse events [4,5]. 125I seed brachytherapy is a treatment method involving the implantation of multiple small capsules containing the radioactive substance Iodine-125 (125I) into the prostate. Naturally, if the source activity of 125I differs from the manufacturer's stated nominal value, it may affect treatment outcomes. Guidelines in Europe and the United States state that verifying the source activity before treating a patient is the responsibility of the medical physicist [6-11]. In Japan, few facilities conduct verification. Reasons cited include inadequate verification equipment, insufficient knowledge of verification procedures, a shortage of personnel to perform verification, and the fact that many sources circulating domestically cannot be re-sterilized. For these reasons, conducting single-seed assays domestically is difficult [12,13]. Therefore, a batch assay capable of measuring multiple radiation sources under sterile conditions has been proposed as an alternative to the single-seed assay. Previous research on batch assays has been sporadically reported [14-18]. At this facility, we calculate the source strength detection rate (detection rate) from measurements using a well-type ionization chamber to verify the accuracy of the source strength. Understanding the characteristics of the measured values is important for their handling. Previous studies have reported verifications for the model STM1251 (C.R. Bard, Murray Hill, NJ) and the OncoSeed model 6711 (General Electric Healthcare, Barrington, IL) [14-16]. However, to our knowledge, there are no reports verifying whether the detection rate of the TheraAgX100 (Theragenics Corporation, Buford, GA), used at our facility, follows a normal distribution. For batch assays, the nominal value must be calculated from the accumulated detection rate at the facility of use. If the detection rate does not follow a normal distribution, using the mean or median from the batch assay as the nominal value is not appropriate. Therefore, verifying the normality of the detection rate at each facility of use is critically important. Furthermore, since the shape of each source product varies slightly, the radiation emitted from I-125 may be affected by absorption and scattering by the silver wire and the outer titanium shell, potentially influencing the detection rate. Therefore, verifying this for TheraAgX100 is highly significant. The objective of this study is to verify the normality of the detection rate based on the detection rate accumulated at our facility to date.

## Materials and methods

**Figure 1:**
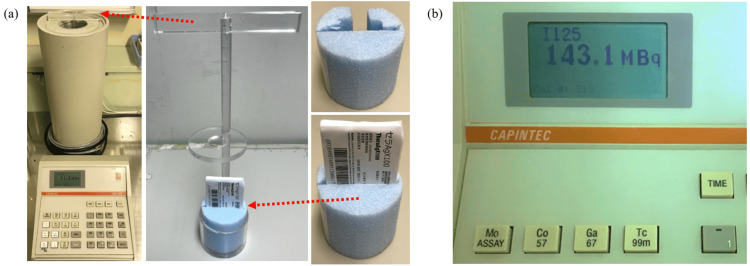
Method for measuring radiation source strength using a well-type ionization chamber (batch assay at our facility). At our facility, we verify source strength using a batch assay with a well-type ionization chamber. The well-type ionization chamber we use is the CRC-15R. As a measurement procedure, we fix the blister pack to a homemade styrofoam pedestal, as shown in image (a). Next, it is attached to an acrylic hanging rod and placed in the well-type ionization chamber. The measured values are then read, as shown in image (b).

At this facility, we verify source intensity using batch assays with a well-type ionization chamber. The well-type ionization chamber used is the CRC-15R (Capintec Corp., Ramsey, NJ). The measurement procedure involves securing the blister pack to a custom-made polystyrene foam base, as shown in Figure 1a. Next, the blister pack is attached to an acrylic suspension rod and placed into the well-type ionization chamber. The measurement value is then read as shown in Figure 1b.

The half-life of 125I is 59.4 days. In busy clinical practice, it is not always possible to perform measurements on the specified date and time. Therefore, when the measurement date differed from the examination date, decay correction was applied to the measured values. In Japan, it is mandatory by law to calibrate measuring instruments regularly. The well-type ionization chamber used in this case was calibrated regularly, and the measured values are reliable. The measurement subjects were the five blister packs with the highest number of deliveries among the delivered TheraAgX100 blister packs, totaling 262 cases. The measurement period was from January 2021 to November 2023. For all of these, the detection rate was calculated from the measurements obtained using a well-type ionization chamber. Normality was verified using Statistical Product and Service Solutions (SPSS, version 29; IBM SPSS Statistics for Windows, Armonk, NY). The Shapiro-Wilk test was used for the test, with a significance level of 0.05.

Following recommendations from the International Atomic Energy Agency, Japan amended the Act on Regulation of Radioactive Isotopes and Related Matters on October 1, 2023, to ensure the reliability of radiation measurements. This amendment requires that “inspection and calibration of radiation measuring instruments be appropriately combined and performed annually.” Compared to the pre-amendment regulations, the law now mandates the use of regularly calibrated dosimeters. The dosimeters used in this study are also regularly calibrated, ensuring the reliability of the measured values.

**Figure 2:**
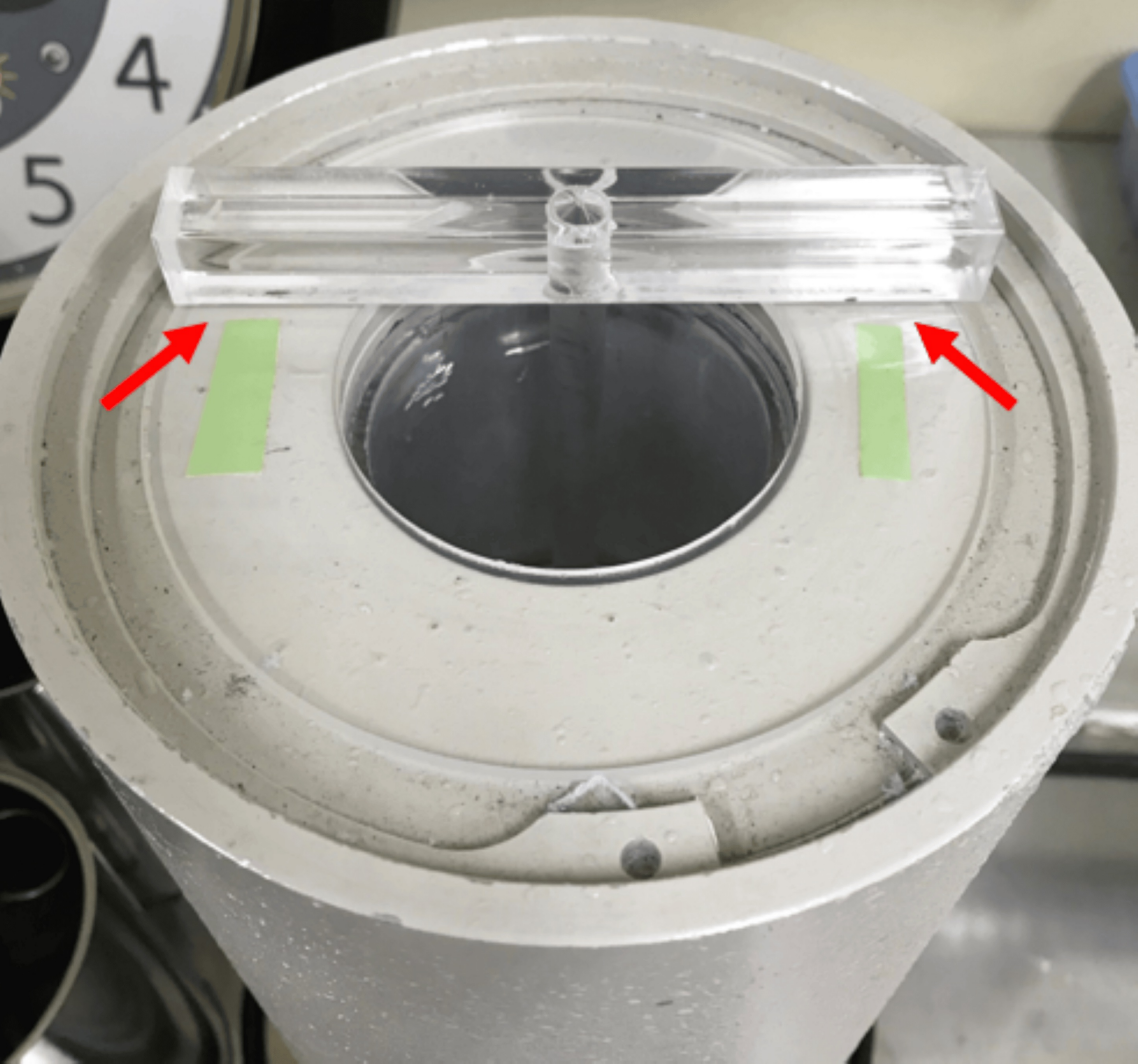
Ensuring measurement reproducibility. As shown in Figure 2, when attaching the suspension rod to the well-type ionization chamber, a mark was applied to ensure it could be positioned in the same location each time. This suppressed variability in each measurement and between operators.

One point of caution during measurement was ensuring reproducibility. As shown in Figure 2, when attaching the suspension rod to the well-type ionization chamber, a mark was applied to ensure it could be positioned in the same location each time. This suppressed variability in each measurement and between operators.

**Figure 3:**
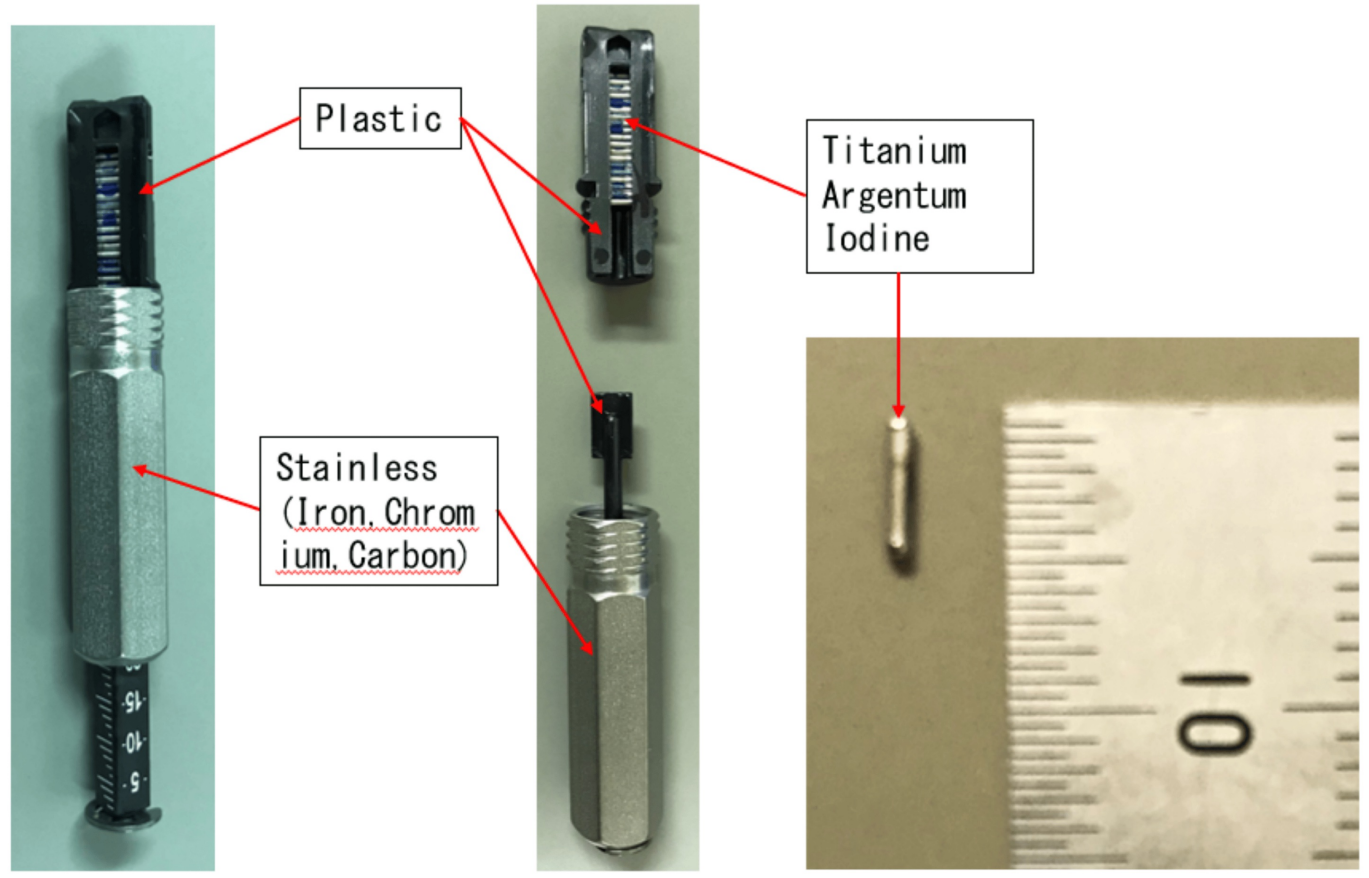
Cartridge and source materials. Figure 3 describes the materials constituting the cartridge enclosed within the blister pack. The cartridge is composed of plastic and stainless steel, while the radiation source consists of titanium, silver, and iodine.

Figure 3 describes the materials constituting the cartridge enclosed within the blister pack. The cartridge is composed of plastic and stainless steel, while the radiation source consists of titanium, silver, and iodine.

**Figure 4:**
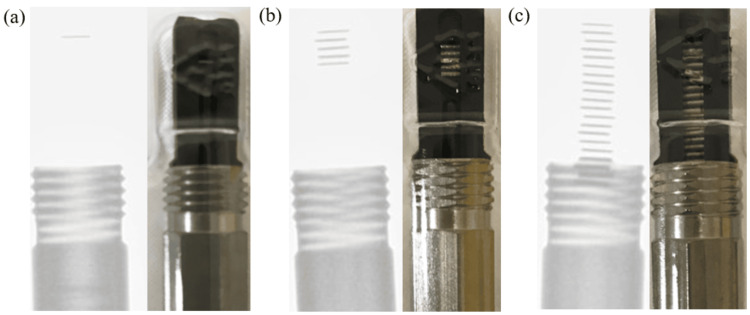
X-ray fluoroscopy image of a blister pack. Figure 4 shows X-ray images of blister packs containing (a) one unit, (b) five units, and (c) 20 units. As seen in the figure, the position of the radiation source varies depending on the number of units. In the case of five units, all sources are located within the plastic portion.

Figure 4 shows the X-ray images of blister packs containing (a) one unit, (b) five units, and (c) 20 units. As seen in the figure, the position of the radiation source varies depending on the number of units. In the case of five units, all sources are located within the plastic portion.

As a preliminary verification for the measurement, the detection rate of the well-type ionization chamber in the depth direction was evaluated. The detection rate sensitivity was 100% for the range where one to twenty blister pack sources were present.

Additionally, to ensure measurement stability, readings were taken 60 seconds after inserting the source into the well-type ionization chamber.

The detection rate calculation formula is as follows:

$Detection Rate (\%)=\frac{Measured Value by Well-Type Ionization Chamber (MBq)}{Manufacturer's Nominal Value (MBq)*Number of Packaged Sources in Blister Pack (units)}*100$

## Results

**Figure 5:**
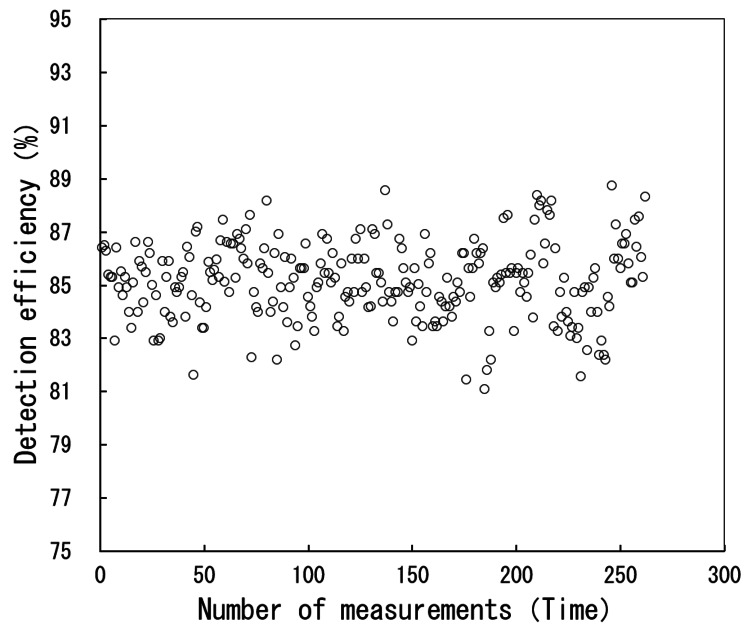
Scatter plot of the detection rate.

**Figure 6:**
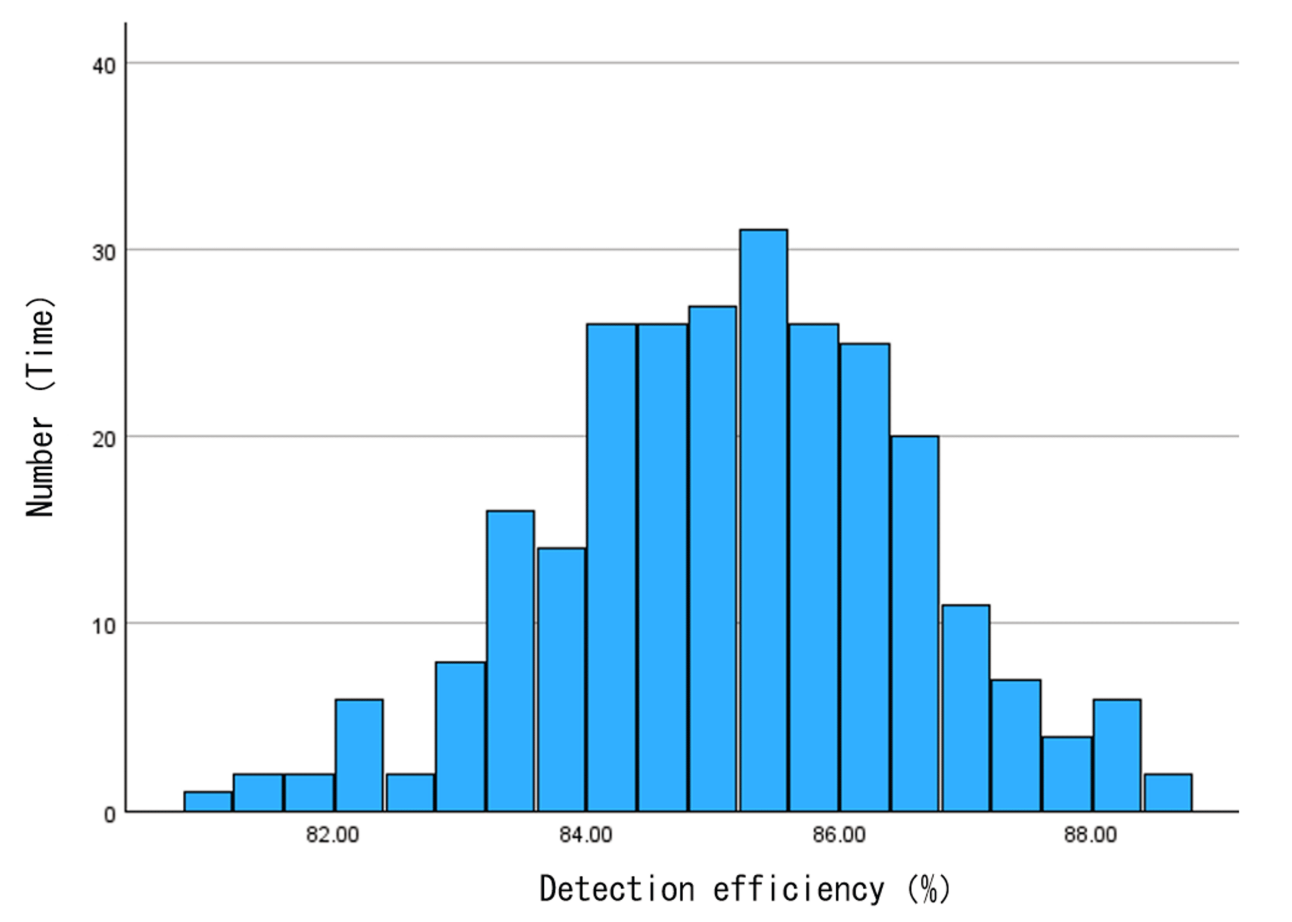
Histogram of the detection rate.

**Figure 7:**
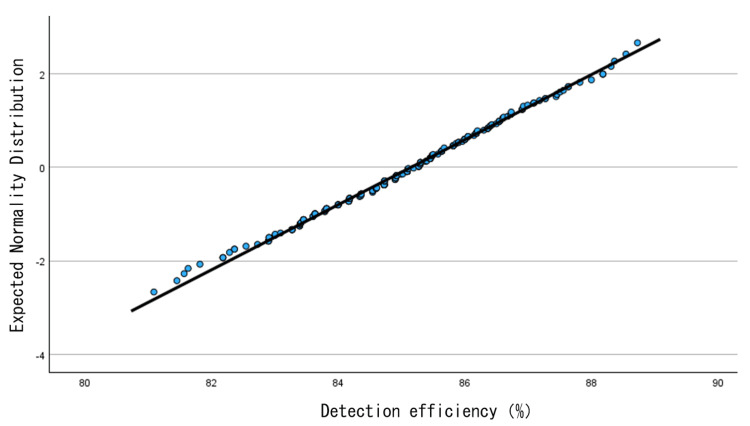
Q-Q plot of the detection rate.

Figure 5 shows the scatter plot of the detection rate. Figure 6 shows the histogram of the detection rate. Figure 7 shows the Q-Q plot of the detection rate. The results of the normality test using SPSS revealed that the Shapiro-Wilk test significance probability was 0.623, confirming that the detection rate follows a normal distribution.

Figure 5 shows that the detection rate plot exhibits a wide distribution. The mean and median detection rates were calculated from all 262 data cases. The mean was 85.1%, and the median was 85.3%. Subsequently, the acceptable limits and intervention limits for the detection rate were calculated from the mean. The tolerance limits, set at the mean ± 3%, corresponded to detection rates of 82.6%-87.7%, encompassing a total of 21 cases. Specifically, nine samples exceeded +3% and 12 samples fell below -3%. The intervention limits, set at the mean ± 5%, corresponded to detection rates of 80.9% to 89.4%, and no cases fell within this range. Figure 7 shows that the plot is linear and follows a normal distribution.

## Discussion

Naturally, in brachytherapy, uncertainty in source intensity directly translates to uncertainty in the prescribed dose. In other words, if the precise source intensity cannot be determined, it may influence treatment outcomes. In 125I seed brachytherapy, this is arguably as important as the physician's source placement technique. Consequently, numerous earlier papers on measuring the strength of 125I sources have been published for quite some time [19-25].

Research on batch assays for 125I seed brachytherapy has been conducted for a long time. Lee et al. found that replacing active seeds with dummy seeds resulted in a charge reduction of approximately 20%, demonstrating that the proposed five-seed assay method can detect dead seeds. They reported that this method can serve as a practical and realistic quality assurance procedure due to its advantages in terms of simple operation, reproducibility, and verification time [26].

Previous studies reported analysis results for two types of single model seeds distributed in Japan, confirming that they match the source intensity published by the manufacturers [14,15]. Furthermore, it has been reported that the batch assay for blister packs closely matches the source intensity published by the manufacturer [16]. The shape and material of the radiation source vary slightly between products, necessitating individual verification of batch assay results. Furthermore, when a manufacturer releases a new product, measurement data must be collected anew to evaluate its characteristics. While prior studies have reported using histograms to assess normality, to our knowledge, no reports exist that statistically evaluate the normality of batch assays using statistical analysis software such as SPSS. An increase in reports similar to this study is expected to contribute to the wider adoption of batch assay validation.

For batch assays, the nominal value must be calculated from the accumulated measurement data at the facility. In other words, without confirming the normality of the detection rate, it is unclear whether the mean or median should be adopted as the nominal value. This study revealed that, for the combination of radiation sources and dosimeters at this facility, the detection rate follows a normal distribution. Therefore, either the mean or the median of the detection rate can be appropriately used as the nominal value.

In a previous paper, Otani et al. reported that none of the OncoSeed model 6711 cartridges exceeded the nominal value by more than 3% [16]. This study revealed that 21 out of 262 cases (8.02%) from five blister packs of TheraAgX100 exceeded the acceptable limit of 3%. This result differs from Otani et al.'s study, suggesting the importance of verifying each source model individually. Furthermore, no samples exceeded the intervention threshold of 5%, indicating that a certain level of quality is assured regarding source strength. Although the source models differ, the findings regarding quality assurance align with previous studies [12,16].

Based on the actual measurement data from this study, no instances were found where the source intensity of TheraAgX100 exceeded the intervention limit of ±5% in batch assays. This indicates that the quality of source intensity for TheraAgX100 is also considered assured. The guidelines recommend that, if the intervention limit is exceeded, “consult with the manufacturer to investigate the cause of the deviation” and “consult with the physician regarding the results obtained when adopting the measured values.” To provide safe medical care to patients, it is considered important for facilities performing 125I seed brachytherapy to thoroughly implement source strength verification in compliance with the guidelines.

In Japan, single-seed assays are difficult from the aforementioned perspective. Therefore, source strength verification must be performed using batch assays. Batch assays enable efficient measurement of all sources while minimizing the exposure dose to the operator.

In 125I seed brachytherapy, the insertion of dead seeds or sources with mismatched certification dates can have significant consequences, making source strength verification a critical task. In Japan, the revision of the Medical Device Safety Management Fee 2 now allows facilities meeting the requirements to bill for this service in seed therapy. The Japanese Society for Radiation Oncology (JASTRO) guidelines were published as specific requirements, recommending source quantity verification and source strength measurement as part of source quality control. Previous reasons cited for the limited adoption of source verification included “staff shortages” and “lack of measurement equipment.” This revision is expected to provide impetus for resolving these issues.

As a limitation of this study, we verified five blister packs using TheraAgX100 and CRC-15R. Similar results cannot be guaranteed depending on the source type, well-type ionization chamber dosimeter, or the number of sources used. Therefore, when adopting a batch assay, verification based on the cumulative detection rate for each facility is necessary. Additionally, measurement uncertainty and reproducibility effects, as well as potential bias arising from sample selection criteria, may occur.

## Conclusions

The guidelines describe the response when batch assay results deviate from the nominal value. Specific control limits are set: a 3% deviation is the tolerance limit, and a 5% deviation is the intervention limit. For batch assays, the nominal value must be calculated based on the cumulative detection rate at the facility. If the detection rate does not follow a normal distribution, using the mean or median from the batch assay as the nominal value is not appropriate. This study demonstrated the validity of using the mean or median of the cumulative detection rate as the nominal value. We plan to verify this approach for blister packs containing different numbers of units. Furthermore, since the shape of the radiation source varies by product, re-evaluation is necessary when new products are introduced. Understanding the characteristics of the acquired measurements a applying this knowledge to clinical practice is a critical responsibility of medical physicists in charge of quality assurance. We aim to continue providing high-precision and safe treatments through ongoing measurements.
